# Protein coated microcrystals formulated with model antigens and modified with calcium phosphate exhibit enhanced phagocytosis and immunogenicity^☆^

**DOI:** 10.1016/j.vaccine.2013.09.061

**Published:** 2014-07-16

**Authors:** Sarah Jones, Catpagavalli Asokanathan, Dorota Kmiec, June Irvine, Roland Fleck, Dorothy Xing, Barry Moore, Roger Parton, John Coote

**Affiliations:** a Institute of Infection, Immunity and Inflammation, College of Veterinary, Medical and Life Sciences, University of Glasgow, University Avenue, Glasgow G12 8QQ, UK; b Division of Bacteriology, National Institute of Biological Standards and Control (NIBSC), Blanche Lane, South Mimms, Hertfordshire EN6 3QG, UK; c Division of Cellular Biology and Imaging, National Institute of Biological Standards and Control (NIBSC), Blanche Lane, South Mimms, Hertfordshire EN6 3QG, UK; d Department of P&A Chemistry, WestChem, Thomas Graham Building, 295 Cathedral Street, Glasgow G1 1XL, UK; e XstalBio Ltd., CIDS, Thomson Building, University Avenue, Glasgow G12 8QQ, UK

**Keywords:** ANOVA, analysis of variance, Block-B, 1% BSA in PBST, Block-G, 1% gelatine in PBST, BSA, bovine serum albumin, BSA-FITC, BSA conjugated to FITC, CaP, calcium phosphate, cRPMI, complete RPMI medium, CyaA, adenylate cyclase toxin of *Bordetella pertussis*, CyaA*, genetically-detoxified CyaA, DAPI, 4′,6-diamidino-2-phenylindole, DT, diphtheria toxoid, DTaP, diphtheria, tetanus and acellular pertussis vaccine, FITC, fluorescein isothiocyanate, PBS, phosphate-buffered saline, PBS-A, PBS lacking Mg^2+^ and Ca^2+^, PBST, PBS containing 0.05% Tween 20, PCMC, protein-coated microcrystal, PVDF, polyvinylidene fluoride, SEM, scanning electron microscopy, rt, room temperature, Microparticles, Calcium phosphate, Phagocytosis, Adjuvant

## Abstract

Protein-coated microcrystals (PCMCs) were investigated as potential vaccine formulations for a range of model antigens. Presentation of antigens as PCMCs increased the antigen-specific IgG responses for all antigens tested, compared to soluble antigens. When compared to conventional aluminium-adjuvanted formulations, PCMCs modified with calcium phosphate (CaP) showed enhanced antigen-specific IgG responses and a decreased antigen-specific IgG1:IgG2a ratio, indicating the induction of a more balanced Th1/Th2 response. The rate of antigen release from CaP PCMCs, *in vitro*, decreased strongly with increasing CaP loading but their immunogenicity *in vivo* was not significantly different, suggesting the adjuvanticity was not due to a depot effect. Notably, it was found that CaP modification enhanced the phagocytosis of fluorescent antigen-PCMC particles by J774.2 murine monocyte/macrophage cells compared to soluble antigen or soluble PCMCs. Thus, CaP PCMCs may provide an alternative to conventional aluminium-based acellular vaccines to provide a more balanced Th1/Th2 immune response.

## Highlights

• Calcium phosphate slows antigen release from protein coated microcrystals.
• Specific IgG titres are enhanced for a number of model antigens.
• Immunogenicity does not correlate with rate of antigen release from depot.
• Th1-biased immunity is higher than with alum adjuvant.
• Phagocytosis is promoted by calcium phosphate modification.

## 1 Introduction

Conventional aluminium-containing adjuvants have been used in vaccine formulations for decades but promote poor induction of Th1 or cell-mediated immunity [1,2] and require refrigeration during transportation and storage. Approximately 50% of vaccines are discarded globally, largely due to cold chain disruption [3,4]. Therefore, a major objective of vaccine formulation *t* is to develop a safe, immunogenic composition which addresses the issues of immune bias and stability.

Protein-coated microcrystals (PCMCs) are a recent advance in vaccine formulation [5] and have the potential to by-pass the cold chain. Originally developed to stabilise enzymes for industrial applications [5–9], PCMCs are formed by rapid co-precipitation of protein(s) with an amino acid or sugar, producing particles with an inert core microcrystal coated with protein(s) [6,8,9]. Vaccine antigens, loaded onto PCMCs, exhibited much higher resistance to heat stress compared to native antigens [5,7]. These reports used PCMC formulations which were instantly soluble in aqueous buffer [5–9]. In this study, novel sustained-release PCMCs have been used which are poorly soluble due to modification of their outer surface with sparingly soluble CaP. CaP served as an adjuvant in some early acellular vaccines [10,11], and is well-tolerated in man [11–16]. CaP also enhances Th1-biased immunity although this may be antigen-dependent [11,17,18]. Here, the immunogenicity of CaP-modified PCMCs loaded with different model antigens was investigated. DT, a formaldehyde-toxoided antigen [19–21], and BSA have been used extensively as model antigens when validating new vaccine formulations [22–25].

## 2 Materials and methods

### 2.1 Source of antigens

The DT preparation was the 2nd international standard for use in flocculation tests (02/176, NIBSC, UK). CyaA* was purified and characterised as described previously [26–28]. BSA was from Sigma and BSA-FITC was from Life Technologies, UK.

### 2.2 PCMC preparation

All reagents were of the highest grade available and were used at rt. The aqueous solution was prepared in endotoxin-free, sterile water (Sigma) and contained 30 mg/ml l-glutamine as the core component of the PCMCs, trehalose and the test antigens, sufficient to give final loadings of 10% and 0.2–0.4%, respectively, in the PCMC preparation. To precipitate PCMCs, 3 ml of the aqueous solution was added drop-wise to 60 ml of rapidly stirred isopropanol and stirring continued for 1 min at 1500 rpm. For CaP-modified PCMCs, the required concentration of NaH_2_PO_4_ was included in the aqueous solution and CaCl_2_ was included in the isopropanol at a 2-fold molar excess compared to NaH_2_PO_4_. PCMCs were collected by vacuum filtration onto PVDF hydrophilic 0.45 μm filters (Millipore, UK) and dried overnight for storage as a dry powder.

### 2.3 Quantification of antigen loading by ELISA

PCMCs were dissolved at 10 mg/ml in sodium citrate buffer [50 mM sodium citrate, 20 mM Tris, 1 mM EDTA, pH6.8]. The PCMC solution was diluted 1:3 v/v in carbonate coating buffer [15 mM Na_2_CO_3_, 30 mM NaHCO_3_, pH9.5] and serially diluted in a flat-bottom 96-well ELISA plate (MAXISorp, Nunc, UK). Plates were incubated overnight at 4 °C prior to washing 3 times in PBST. Non-specific binding was blocked by addition of 100 μl/well of block-B and incubation for 1 h at 37 °C. For BSA-containing PCMCs, block-G was used in place of block-B. After further washing, samples were incubated (2 h, 37 °C) with 50 μl/well of the appropriate primary antibody [anti-DT (NIBSC, 1/1000), anti-CyaA* (in-house, 1/500)] or anti-BSA (Sigma, 1/1000)] diluted in the appropriate blocking buffer. After washing, 50 μl/well of peroxidase-conjugated secondary antibody (Sigma) diluted 1/1000 in the appropriate blocking buffer was added and plates incubated for 1.5 h at 37 °C. Plates were washed again and protein binding was visualised using 50 μl/well of O-phenylene-diamine. After incubation for 10–15 min at rt, colour development was stopped with 3 M HCl and absorbance at 492 nm was measured. Protein loading onto PCMCs was quantified by comparison to a stock antigen standard curve.

### 2.4 Determination of PCMC morphology

For SEM, dry PCMCs were gold-plated prior to visualisation with a JEOL6400 electron microscope operating at 6 kV.

### 2.5 Characterisation of antigen release *in vitro*

PCMCs were suspended at 10 mg/ml in 1.5 ml of either 0.1 mM sodium citrate (pH 6.0) or PBS and incubated at rt or 37 °C with gentle agitation. At intervals, the PCMC suspension was centrifuged for 1 min at 2400 × *g* and 1 ml of supernate removed to determine protein release. More buffer was then added to the pelleted PCMCs to readjust the volume to 1.5 ml and the incubation continued. Supernates were stored at −20 °C prior to quantification of protein release by ELISA as described above.

### 2.6 Adsorption of antigens to Al(OH)_3_

Soluble antigens were dissolved in sterile PBS containing 10% Al(OH)_3_ (A8222, Sigma), mixed thoroughly and incubated overnight at 4 °C. Adsorbed antigens were then used for immunisation.

### 2.7 Immunisation of mice

Groups of 8 inbred, female 6–8 week old NIH mice (Harlan, UK) were injected subcutaneously at days 0 and 28 with 0.5 ml volumes of the desired formulation or PBS as a control. Immediately prior to immunisation, the required doses of PCMCs were suspended in sterile PBS. Mice were sampled for sera at 28 d and 42 d post-immunisation, as described previously [28]. All animal experiments were performed under UK Home Office License and in accordance with EU Directive 2010/63/EU.

### 2.8 Determination of antigen-specific serum IgG titres

Antigen-specific IgG, IgG1 and IgG2a titres were determined using ELISA as described previously [26] with the use of block-G when determining anti-BSA responses. Geometric mean titres were calculated by comparison to reference sera.

### 2.9 Culture of J774.2 cells

Murine monocyte/macrophage J774.2 cells were maintained in 75 cm^2^ tissue-culture flasks (Corning, UK) (37 °C, 5% CO_2_) in complete RPMI [cRPMI; RPMI 1640 medium (Life Technologies, UK), 10% foetal calf serum (Sigma), 10 mM l-glutamine, 100 U/ml penicillin and 100 μg/ml streptomycin (Life Technologies, UK)].

### 2.10 Uptake of PCMCs by J774.2 cells

#### 2.10.1 Flow cytometry

Each well of a 24-well tissue-culture plate (Corning, UK) was supplemented with 10^6^ J774.2 cells and incubated (2 h, 37 °C, 5% CO_2_) after which the medium was replaced with 1 ml/well of fresh cRPMI. A 5 mg/ml suspension of 0–20% CaP PCMCs loaded with 0.4% BSA-FITC or the equivalent concentration of soluble BSA-FITC were prepared in cRPMI. A 0.5 ml aliquot was added to each well and incubated (1 h, 37 °C, 5% CO_2_) whilst protected from light. To stop uptake, cells were washed twice with ice-cold PBS and suspended in 1 ml of ice-cold PBS. Cells were centrifuged for 10 min at 118 × *g*, the resultant pellet suspended in 4 ml of fixing solution [1% formaldehyde in PBS] and samples stored at 4 °C whilst protected from light. Uptake of fluorescent particles was determined using a FACSCanto II flow cytometer (BD Biosciences).

#### 2.10.2 Confocal laser-scanning microscopy

Sterile glass coverslips were coated with 0.2% gelatine in PBS and air-dried. An aliquot of 10^6^ J774.2 cells in 2 ml of cRPMI was added to each well (24-well tissue-culture plate) containing coated coverslips and incubated (3 h, 37 °C, 5% CO_2_) for cell attachment. Cells were then incubated (1 h, 37 °C, 5% CO_2_) with the appropriate antigen formulation and washed twice with PBS-A, then fixed (300 μl/well, 4% paraformaldehyde in PBS-A) and incubated (20 min, rt). Cells were permeabilised by incubation with PBS-A containing 0.2% BSA and 0.2% Triton X-100 and secondary incubation with PBS-A containing 5% BSA. After washing, the actin cytoskeleton was stained with AlexaFluor594-conjugated phalloidin (Life Technologies, UK) for 5 min prior to nuclear staining with 4′,6-diamidino-2-phenylindole (DAPI) for 3 min. After washing, the coverslips were mounted onto glass microscope slides and cell fluorescence visualised using a Leica SP2 AOBS laser-scanning confocal microscope (40×, NA 1.25 oil immersion lens). Images were analysed using IMARIS software v7.4.2 (Bitplane, Switzerland).

### 2.11 Statistical analysis

Statistical analysis was performed using GraphPad Prism5 software. Gaussian distribution of the data was assessed using the D’Agostino and Pearson omnibus normality test. Responses between several groups were compared by one-way analysis of variance (ANOVA) with Tukey's, Bonferroni's or Dunn's correction, as appropriate. Where data failed to pass the normality test, non-parametric comparison between several groups was by the Kruskal–Wallis test. Comparison of data between two groups was performed using Student's *t*-test. Statistical significance was defined as *p* < 0.05.

## 3 Results

### 3.1 Inclusion of CaP alters PCMC morphology and significantly decreases antigen release rate *in vitro*

**Fig. 1:**
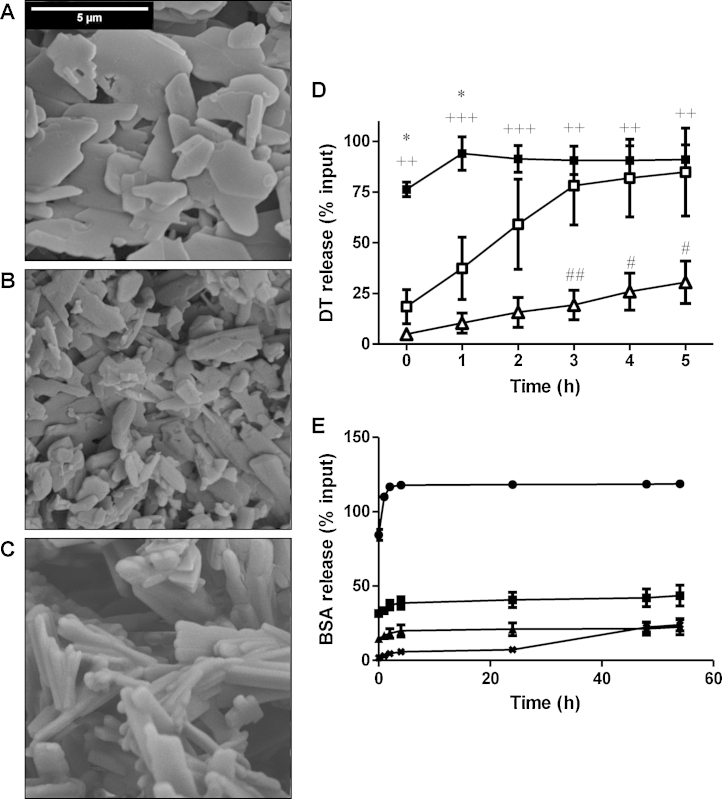
Effect of CaP on the morphology of PCMCs and the rate of antigen release. Panels A–C: PCMCs were loaded with 0.2% (w/w) DT and either 0% CaP (panel A), 6% CaP (panel B) or 20% CaP (panel C). Dried PCMC preparations were gold-plated and visualised by SEM at 5000 × magnification. Scale bar = 5 μm. Images are representative of at least *n* = 2 independent SEM preparations. Panel D: PCMCs were prepared with 0.2% loading of each of DT and CyaA* and resuspended at 10 mg/ml in 1 mM sodium citrate, pH 6.0 at room temperature with gentle agitation. Samples were taken at 1 h intervals and the protein release quantified by ELISA for 0% CaP (closed squares), 6% CaP (open squares) and 20% CaP (triangles) PCMCs. *p < 0.05 0% CaP *vs.* 6% CaP PCMCs, ^++^*p* < 0.01 0% CaP *vs.* 20% CaP PCMCs, ^+++^*p* < 0.001 0% CaP *vs.* 20% CaP PCMCs, ^#^*p* < 0.05 6% CaP *vs.* 20% CaP PCMCs, ^##^*p* < 0.01 6% CaP *vs.* 20% CaP PCMCs. Panel E: PCMCs were prepared with 0.4% loading of BSA-FITC and resuspended at 10 mg/ml in sterile PBS at 37 °C with gentle agitation. Samples were taken at intervals and BSA-FITC release in the supernatant determined by ELISA for 0% CaP (squares), 6% CaP (circles), 12% CaP (triangles) and 20% CaP PCMCs (crosses). Results are representative triplicate measurements of at least *n* = 3 independent experiments.

SEM showed that soluble PCMCs loaded with antigen without CaP (0% CaP PCMCs) were planar, irregular discs (Fig. 1A) but, as the CaP loading increased, the particles became more regular rod-like structures (Fig. 1B and C). This change in morphology was antigen-independent over the 0.2–0.4% antigen loading used (not shown).

The *in vitro* release of antigen from different CaP PCMC formulations was compared by suspending the particles in 0.1 mM sodium citrate, pH 6.0 at rt. PCMCs without CaP and loaded simultaneously with DT and CyaA* released DT almost instantaneously whilst the 6% and 20% CaP PCMCs displayed progressively delayed antigen release (Fig. 1D). Similar results were obtained for all antigens and combinations tested, indicating that the phenomenon was not antigen-specific (not shown). BSA-FITC release from PCMCs suspended in PBS at 37 °C was investigated as a more physiologically relevant model. BSA-FITC release from PCMCs without CaP was extremely rapid but was significantly slower with CaP PCMCs (Fig. 1E).

### 3.2 PCMCs are more immunogenic than soluble antigen alone

**Fig. 2:**
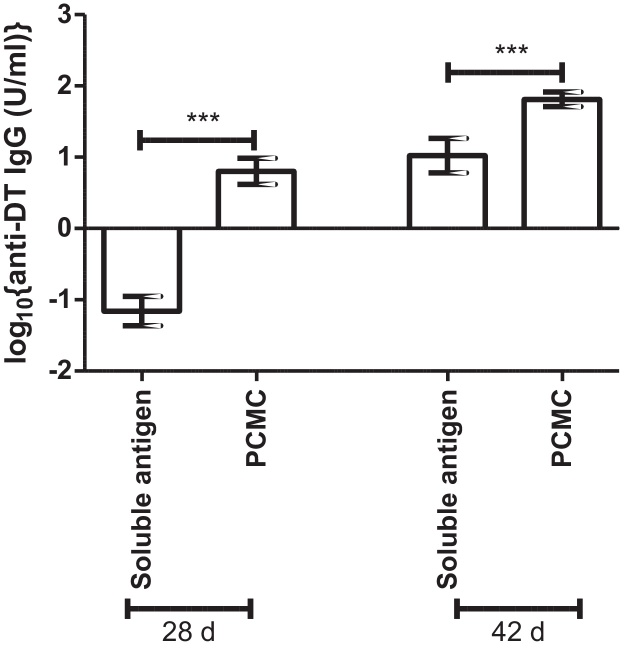
Effect of PCMC formulation on the immunogenicity of soluble antigens. 8 mice/group were immunised subcutaneously with 12 μg/dose DT at 0 d, administered as either PCMCs or soluble antigen prior to boosting at 28 d. Anti-DT IgG titres were determined by ELISA in serum taken at 28 d and 42 d post-immunisation. Data represent mean log_10_{geometric mean anti-DT IgG titre (IU/ml)} ± SEM for *n* = 8 mice/group ***p < 0.001. Results are representative of *n* ≥ 2 independent experiments.

Subcutaneous injection of mice with PCMCs loaded with DT in the absence of CaP induced significantly higher anti-DT IgG titres than the equivalent soluble antigen at both 28 d and 42 d (Fig. 2). Similar effects were seen with the other antigens indicating that this response was not antigen-specific (data not shown).

### 3.3 CaP modification increases the immunogenicity of antigens loaded onto PCMCs

**Fig. 3:**
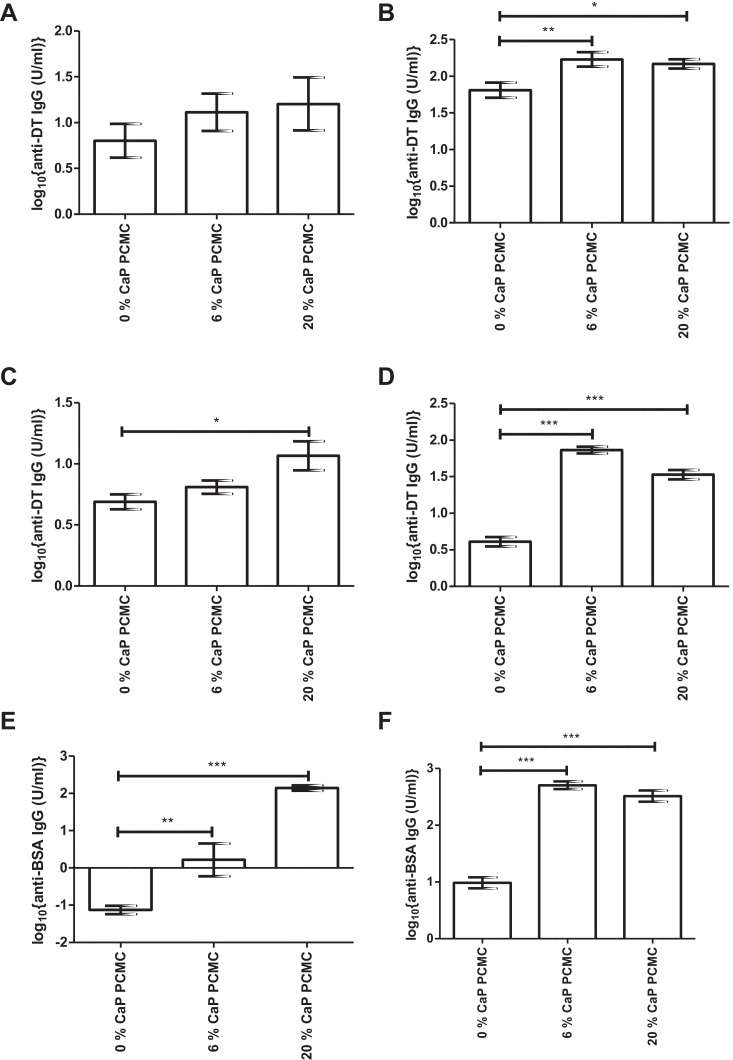
Effect of PCMC formulation on serum antigen-specific IgG responses. Panels A–D: 8 mice/group were immunised subcutaneously with 12 μg/dose DT formulated as 0%, 6% or 20% CaP PCMCs in the absence (panels A and B) or presence of CyaA* (panels C and D) at 0 d and boosted with equal doses at 28 d. Serum anti-DT IgG responses were determined at 28 d (panels A and C) and 42 d (panels B and D) post-immunisation by ELISA. Panels E and F: 8 mice/group were immunised subcutaneously with 8 μg/dose BSA formulated as 0%, 6% or 20% CaP PCMCs at 0 d and boosted with equal doses at 28 d. Serum anti-BSA IgG responses were determined at 28 d (panel E) and 42 d (panel F) post-immunisation by ELISA. Data represent mean log_10_{geometric mean antigen-specific IgG titres} ± SEM for *n* = 8 mice/group *p < 0.05, **p < 0.01, ***p < 0.001. Results are representative of *n* ≥ 2 independent experiments.

Whilst formulation into PCMCs enhanced the immune response to DT, it was likely that surface modification with CaP would further enhance antigen-specific IgG titres. Mice were immunised with 0%, 6% or 20% CaP PCMCs loaded with DT, DT + CyaA* or BSA. CaP PCMCs enhanced the antigen-specific IgG response to DT and BSA at 28 d and 42 d post-immunisation (Fig. 3). For PCMCs loaded with DT alone, CaP modification increased serum anti-DT IgG titres prior to boosting (Fig. 3A) but the effect was more pronounced after boosting (Fig. 3B). Inclusion of CyaA* did not alter the adjuvant effect of CaP on the anti-DT IgG response at 28 d (Fig. 3C) and 42 d (Fig. 3D). The adjuvant activity of CaP was not confined to DT, as CaP PCMCs also promoted an increase in anti-BSA IgG titres at 28 d (Fig. 3E) and 42 d (Fig. 3F).

### 3.4 CaP PCMCs alter the antigen-specific Th1/Th2 response

**Fig. 4:**
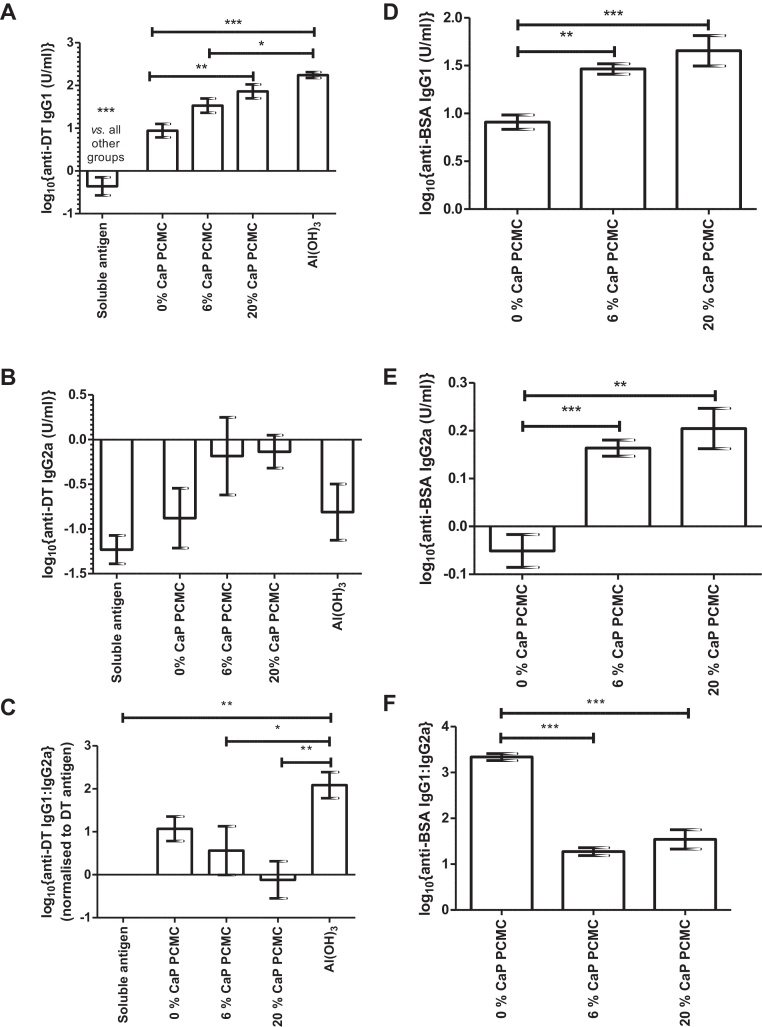
Effect of CaP loading on serum antigen-specific IgG1:IgG2a ratios. Panels A–C: 8 mice/group were immunised subcutaneously with DT 12 μg/dose formulated as soluble antigen, 0% CaP PCMCs, 6% CaP PCMCs, 20% CaP PCMCs or adsorbed to Al(OH)_3_ and boosted with equal doses at 28 d. Serum anti-DT IgG1 (panel A) and IgG2a titres (panel B) were determined at 42 d post-immunisation by ELISA. The IgG1:2a ratios for matched serum samples were also determined (panel C). Panels D–F: 8 mice/group were immunised subcutaneously with 8 μg/dose BSA formulated as 0% CaP PCMCs, 6% CaP PCMCs or 20% CaP PCMCs and boosted with equal doses at 28 d. Serum anti-BSA IgG1 (panel D) and IgG2a (panel E) responses were determined at 42 d post-immunisation by ELISA. The IgG1:2a ratios for matched serum samples were also determined (panel F). Data represent mean log_10_{geometric mean antigen-specific IgG titres} ± SEM for *n* = 8 mice/group *p < 0.05, **p < 0.01, ***p < 0.001. Results are representative of *n* ≥ 2 independent experiments.

Serum antigen-specific IgG1 and IgG2a titres were determined in order to assess whether CaP modification altered the Th1/Th2 bias. In mice, a decreased IgG1:IgG2a ratio is associated with a Th1-biased immune response [29]. Adsorption of DT to Al(OH)_3_ resulted in a high IgG1 response (Fig. 4A) and a high anti-DT IgG1:IgG2a ratio (Fig. 4C) compared to soluble antigen or PCMC formulations_._ Increasing CaP loading increased both the anti-DT IgG1 and IgG2a titres (Fig. 4A and B) but the overall effect was to decrease the anti-DT IgG1:IgG2a ratio (Fig. 4C). Modification with CaP significantly increased the anti-BSA IgG1 and IgG2a titres (Fig. 4D and E) but decreased the anti-BSA IgG1:IgG2a ratio compared to soluble (0% CaP) PCMC formulations (Fig. 4F).

### 3.5 CaP loading does not affect the duration or magnitude of the antibody responses

The results above demonstrated that CaP modification had an adjuvant effect on PCMC-induced antigen responses *in vivo*, although increasing the CaP loading from 6 to 20% did not have a significantly consistent dose-dependent effect. To investigate this further, mice were immunised with a single dose of 0%, 6%, 12% or 20% CaP PCMCs loaded with 6 μg/dose each of DT and CyaA* and the kinetics of the serum antigen-specific IgG responses determined up to 84 d post-immunisation. Mice immunised with equal amounts of 6% and 20% CaP PCMCs were also included to investigate any prime/boost effect arising from fairly rapid antigen release from 6% CaP PCMCs and a more prolonged depot effect of 20% CaP PCMCs. The adjuvant effect of including CaP in PCMCs was confirmed for both antigens (Table 1). This was particularly marked for the anti-CyaA* response as only one mouse in the 0% CaP group produced a detectable anti-CyaA* IgG titre at each time point investigated. Increasing the CaP content did not significantly further increase the antigen-specific IgG titres or alter the duration of antibody response. The attempted prime-boost formulation failed to enhance immunogenicity compared to other CaP PCMC formulations.

### 3.6 CaP modification promotes phagocytosis of PCMCs

**Fig. 5:**
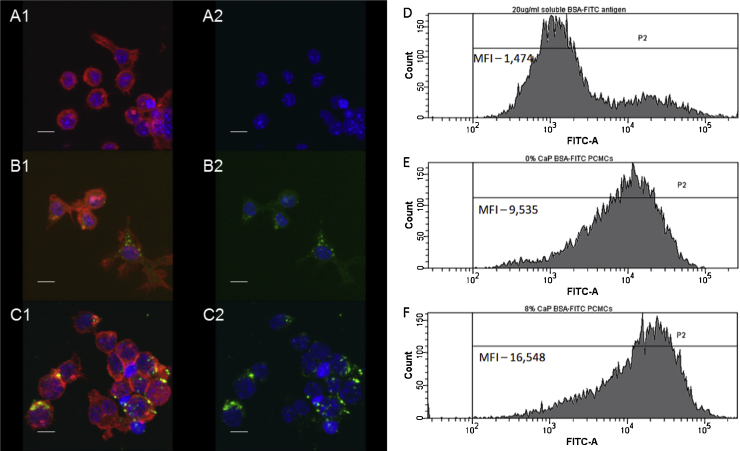
Effect of CaP modification on phagocytosis by macrophages. The figure is a representative result for confocal laser-scanning microscopy and flow cytometry of *n* ≥ 2 independent experiments. J774.2 cells were incubated with 20 μg/ml soluble BSA-FITC antigen (panels A and D), 0% CaP BSA-FITC PCMCs (panels B and E) and 8% CaP BSA-FITC PCMC (panels C and F) for 1 h at 37 °C in an atmosphere of 5% (v/v) CO_2_. Cells treated under identical conditions without incubation with antigen were used as negative controls in all experiments. Uptake of fluorescent antigen was visualised by confocal laser-scanning microscopy (panels A–C, scale bars = 10 μm) and quantified by flow cytometry (panels D–F). Confocal image stacks of each sample were collected for individual emission/detection channels and a composite image formed from data from multi-channels. Images were analysed using IMARIS software v7.4.2 (Bitplane, Switzerland) [green, target protein; blue, nucleus; and red, F-actin of cytoskeleton]. For clarity, each plate is presented showing red/blue/green fluorescence (A1-C1) and also as only blue/green fluorescent channels (A2-C2). The flow cytometry (panels D–F) shows the mean fluorescence intensity (MFI) of the P2 daughter population derived from a live cell gated parent population (P1). (For interpretation of the references to color in figure legend, the reader is referred to the web version of the article.)

J774.2 cells were incubated with equal amounts of either soluble BSA-FITC or BSA-FITC formulated as 0% or 8% CaP PCMCs. Uptake of fluorescent antigen was visualised by confocal laser-scanning microscopy (Fig. 5, panels A–C) and quantified by flow cytometry (panels D–F). Confocal microscopy showed that soluble BSA-FITC was poorly phagocytosed, with J774.2 cells containing low levels of fluorescence (Fig. 5A). In contrast, loading BSA-FITC onto PCMCs increased phagocytosis, with cells displaying punctate regions of green fluorescence (Fig. 5B) and this was further enhanced with CaP PCMCs (Fig. 5C). These observations were confirmed by flow cytometry. The P2 daughter population was derived from the parent population P1. The increase in MFI of the P2-gated population of the cells upon exposure to BSA-FITC PCMCs (Fig. 5E) and the further increase in the presence of CaP-modified PCMCs (Fig. 5F) indicates a greater phagocytosis of these particles compared to soluble BSA-FITC (Fig. 5D).

## 4 Discussion

These results, in combination with published data, demonstrate that PCMC formulations are suitable for vaccine applications and may address problems associated with current vaccines. Moreover, CaP PCMCs were shown to be immunogenic and to promote a more mixed Th1/Th2 response in comparison to traditional formulations and to soluble PCMCs [5,7].

Modification of the surface of PCMC with an outer layer of CaP altered the particle morphology from planar discs to rod-like structures and significantly decreased the rate of antigen release *in vitro*. PCMCs without CaP released antigen almost immediately in aqueous buffers whereas increasing the CaP loading progressively decreased the rate of antigen release. This is consistent with release being controlled by dissolution of an outer layer of CaP, the thickness of which is expected to increase with CaP loading. This suggests that CaP PCMCs would potentially show enhanced immunogenicity due to a depot effect *in vivo* as has been proposed for other adjuvants [2,15].

Surprisingly, mice immunised with DT formulated into soluble PCMCs showed enhanced immunogenicity compared to soluble DT antigen. The *in vitro* solubility data indicated that this enhanced immunogenicity was not due to a depot effect. Instead it may be due to the high local concentration of l-glutamine arising from solubilisation of the PCMC core, since l-glutamine has been shown to enhance immune function [30–33]. Consistent with published data [10,11,17,34], CaP acted as an adjuvant in this study and significantly enhanced CaP PCMC-induced antigen-specific IgG titres compared to soluble PCMCs. The adjuvant effect of CaP and aluminium-based adjuvants has been attributed to their antigen depot effect [2,15]. However, the rate of antigen release from CaP PCMCs had no significant effect on the magnitude or duration of the antibody response and corroborates a growing body of evidence that the activity of traditional adjuvants is independent of a depot effect [35–37]. It should be noted that no significant decrease in antigen-specific IgG titre was observed for any formulation tested up to 84 d post-immunisation. However investigation of the antibody response for longer time periods might highlight differences between the different formulations. CaP PCMC promoted a decrease in antigen-specific IgG1:IgG2a ratio compared to Al(OH)_3_, indicating a more mixed Th1/Th2 immune response. Similar results have been obtained in other studies as a result of both CaP inclusion [17,38] and formulation into microparticle vaccines [39–41].

As the adjuvant effect arising from surface modification of PCMC with CaP was independent of CaP loading, we hypothesised that the morphology of CaP PCMCs may be important for their adjuvant activity. PCMCs are of suitable size and morphology to be phagocytosed by immune cells [42] and phagocytosis of latex microspheres by monocytes promotes their differentiation to functional dendritic cells and subsequent immune priming in the draining lymph node [43]. Formulation into PCMCs without CaP enhanced phagocytosis of BSA-FITC by J774.2 cells, possibly due to enhanced cell function arising from the l-glutamine released from the core component of the soluble PCMCs [30–33]. However, the phagocytosis of BSA-FITC was clearly further enhanced by formulation into CaP PCMCs. Thus, CaP PCMCs may exert their adjuvant effect, at least in part, through enhanced uptake of antigen by tissue phagocytes and subsequent enhancement of immune priming. However, further studies are needed to determine the precise mechanism by which CaP PCMCs exert their adjuvant effect *in vivo*.

Combined with published data [5,7], our results indicate that CaP PCMCs represent a useful platform by which to progress future vaccine formulation.

## Authors’ contributions

SJ performed PCMC preparation, SEM analysis and determination of antigen-specific IgG, IgG1 and IgG2a titres pertaining to PCMCs loaded with DT, CyaA* and BSA. CA performed all *in vivo* experiments. DK prepared PCMCs loaded with BSA-FITC, analysed PCMC uptake by flow cytometry and stained cells for CLSM. JI performed preparation of PCMCs and determined *in vitro* release of DT, CyaA* and BSA release and antigen-specific IgG1 and IgG2a titres. RF captured all CLSM images and prepared them for publication. DX, BM, RP and JGC conceived, co-ordinated, designed and procured the funding for the study. All authors have read and approved the final article.

## Figures and Tables

**Table 1:** Effect of CaP loading on the duration and magnitude of serum antigen-specific IgG responses 8 mice/group were immunised with a single 6 μg/dose each of DT and CyaA* formulated as either 0% CaP PCMCs, 6% CaP PCMCs, 12% CaP and 20% CaP PCMCs or adsorbed to Al(OH)_3_. Serum samples were taken at 28, 42, 63 and 84 d post-immunisation and geometric mean antigen-specific IgG titres (GMT) determined by ELISA. Data show the mean log_10_(geometric mean antigen-specific IgG titres) (±SEM) for *n* = 8 mice/group.

Preparation of antigens as	Log_10_{geometric mean anti-DT IgG titre (U/ml)}				Log_10_{geometric mean anti-CyaA* IgG titre (U/ml)}			
	28 d	42 d	63 d	84 d	28 d	42 d	63 d	84 d
0% CaP PCMC	−2.358 (±0.1846)	−2.369 (±0.285)	−2.975 (±0.313)	−2.895 (±0.231)	−1.126 (±0.272)	−1.187 (±0.211)	−1.220 (±0.178)	−1.220 (±0.178)
6% CaP PCMC	−1.384 (±0.147)^b^	−1.533 (±0.160)^c^	−1.426 (±0.206)^c^	−1.591 (±0.227)^c^	0.009 (±0.259)^b^	0.073 (±0.256)^c^	−0.217 (±0.374)^b^	0.046 (±0.348)^c^
12% CaP PCMC	−1.820 (±0.246)^b^	−1.641 (±0.200)^b^	−1.909 (±0.258)^b^	−2.074 (±0.249)^a^	0.243 (±0.196)^b^	0.066 (±0.163)^a^	−0.215 (±0.250)	0.328 (±0.076)^b^
20% CaP PCMC	−1.246 (±0.203)^c^	−1.296 (±0.189)^c^	−1.456 (±0.189)^c^	−1.617 (±0.189)^c^	0.640 (±0.202)	0.649 (±0.210)	0.614 (±0.227)	0.293 (±0.196)
6% + 20% CaP PCMC	−1.359 (±0.157)^c^	−1.397 (±0.186)^c^	−1.621 (±0.235)^c^	−1.931 (±0.249)^b^	0.184 (±0.410)	0.458 (±0.294)	0.348 (±0.316)	0.120 (±0.374)
Adsorbed to Al(OH)_3_	0.057 (±0.073)^d^	0.113 (±0.063)^d^	0.061 (±0.078)^d^	−0.086 (±0.062)^d^	0.577 (±0.475)	0.780 (±0.421)	0.453 (±0.470)	0.330 (±0.427)

a *p* < 0.05 *vs.* 0% CaP PCMC.

b *p* < 0.01 *vs.* 0% CaP PCMCs.

c *p* < 0.001 *vs.* 0% CaP PCMCs.

d *p* < 0.001 *vs.* all other formulations.
